# Lipopolysaccharide-induced blood-brain barrier disruption: roles of cyclooxygenase, oxidative stress, neuroinflammation, and elements of the neurovascular unit

**DOI:** 10.1186/s12974-015-0434-1

**Published:** 2015-11-25

**Authors:** William A. Banks, Alicia M. Gray, Michelle A. Erickson, Therese S. Salameh, Mamatha Damodarasamy, Nader Sheibani, James S. Meabon, Emily E. Wing, Yoichi Morofuji, David G. Cook, May J. Reed

**Affiliations:** Geriatric Research Education and Clinical Center-VA Puget Sound Health Care System, Seattle, WA USA; Division of Gerontology and Geriatric Medicine, Department of Internal Medicine, University of Washington School of Medicine, Seattle, WA USA; University of Washington School of Medicine, Seattle, WA USA; Ophthalmology and Visual Sciences, University of Wisconsin School of Medicine and Public Health, Madison, WI USA; Mental Health Research Education and Clinical Center-VA Puget Sound Health Care System, Seattle, WA USA; Department of Neurosurgery, University of Nagasaki, Nagasaki, Japan

**Keywords:** Blood-brain barrier, Neurovascular unit, Neuroinflammation, Brain endothelial cells, Indomethacin, Lipopolysaccharide

## Abstract

**Background:**

Disruption of the blood-brain barrier (BBB) occurs in many diseases and is often mediated by inflammatory and neuroimmune mechanisms. Inflammation is well established as a cause of BBB disruption, but many mechanistic questions remain.

**Methods:**

We used lipopolysaccharide (LPS) to induce inflammation and BBB disruption in mice. BBB disruption was measured using ^14^C-sucrose and radioactively labeled albumin. Brain cytokine responses were measured using multiplex technology and dependence on cyclooxygenase (COX) and oxidative stress determined by treatments with indomethacin and N-acetylcysteine. Astrocyte and microglia/macrophage responses were measured using brain immunohistochemistry. In vitro studies used Transwell cultures of primary brain endothelial cells co- or tri-cultured with astrocytes and pericytes to measure effects of LPS on transendothelial electrical resistance (TEER), cellular distribution of tight junction proteins, and permeability to ^14^C-sucrose and radioactive albumin.

**Results:**

In comparison to LPS-induced weight loss, the BBB was relatively resistant to LPS-induced disruption. Disruption occurred only with the highest dose of LPS and was most evident in the frontal cortex, thalamus, pons-medulla, and cerebellum with no disruption in the hypothalamus. The in vitro and in vivo patterns of LPS-induced disruption as measured with ^14^C-sucrose, radioactive albumin, and TEER suggested involvement of both paracellular and transcytotic pathways. Disruption as measured with albumin and ^14^C-sucrose, but not TEER, was blocked by indomethacin. N-acetylcysteine did not affect disruption. In vivo, the measures of neuroinflammation induced by LPS were mainly not reversed by indomethacin. In vitro, the effects on LPS and indomethacin were not altered when brain endothelial cells (BECs) were cultured with astrocytes or pericytes.

**Conclusions:**

The BBB is relatively resistant to LPS-induced disruption with some brain regions more vulnerable than others. LPS-induced disruption appears is to be dependent on COX but not on oxidative stress. Based on in vivo and in vitro measures of neuroinflammation, it appears that astrocytes, microglia/macrophages, and pericytes play little role in the LPS-mediated disruption of the BBB.

## Background

The vascular blood-brain barrier (BBB) protects the central nervous system from blood-borne substances that are otherwise neurotoxic. Inflammation has long been known to disrupt the BBB. Skoog in 1937 found that an allergic reaction could disrupt the BBB [1]. Meningitis, encephalitis, sepsis, and local and systemic infections have been associated with altered permeability of the BBB to many substances and immune cells [2]. Eckman et al. as early as 1958 and Allen a few years later showed that gram-negative endotoxin was associated with BBB disruption [3, 4]. Gram-negative endotoxin in its more purified form of lipopolysaccharide (LPS) is known to disrupt the BBB and also alters many other aspects of BBB function, including adsorptive transcytosis, immune cell trafficking, and various transport functions [5–9]. More recently, a number of diseases that include multiple sclerosis, Alzheimer’s disease, diabetes mellitus, obesity, and stroke have been associated with both inflammation and BBB disruption [10–14].

The barrier functions of the capillary bed of the brain are known to arise from three modifications: the presence of tight junctions between endothelial cells, a near absence of macropinocytosis, and loss of fenestrae [15, 16]. Together, these three modifications prevent the production of an ultrafiltrate by the brain’s capillary bed. Inflammation can induce BBB disruption by altering tight junction function, thus promoting paracellular leakage, and re-inducing vesicular processes, thus promoting transcytotic leakage. In some causes, transcytotic mechanisms may be the dominant form of BBB disruption [17, 18] and may precede paracellular opening [19, 20].

Mechanisms by which LPS affects BBB function are not well understood. The cyclooxygenase (COX) inhibitor indomethacin reverses some LPS-induced dysfunctions of the BBB, including in vitro disruption [21, 22] and in vivo impaired amyloid beta peptide efflux [23]. However, indomethacin has no effect on LPS-induced inhibition of P-glycoprotein activity [24]. The antioxidant N-acetylcysteine (NAC) protects the brain endothelial cell (BEC) from oxidative stress induced by methamphetamine and antiretroviral drugs [25, 26] and reverses the decreased brain-to-blood transport of amyloid beta peptide induced by LPS [27]. The effect of NAC on LPS-induced BBB disruption has not been examined in vivo.

Here, we further examined the mechanisms by which LPS induces BBB disruption both in vivo and in vitro by investigating brain cytokines, prostaglandin inhibition, oxidative stress, and astrocyte and microglial activation.

## Methods

### Animals

All mice were treated in accordance with NIH Guidelines for the Care and Use of Laboratory Animals in an AAALAC-accredited facility and approved by the Institutional Animal Care and Use Committee of the VA Puget Sound Health Care System. Male CD-1 mice at 6–8 weeks of age were purchased from Charles River and kept on a 12/12-h light/dark cycle with ad libitum food and water.

### Radioactive probes

Sucrose labeled with ^14^C was purchased from Perkin-Elmer (Waltham, MA). Bovine serum albumin (BSA) was labeled with ^125^I by the chloramine-T method, and the resulting radioactive albumin (iodine-labeled albumin (I-Alb)) was purified on a G-10 Sephadex column. BSA was labeled with ^99m^Tc (technetium-labeled albumin (Tc-Alb)) and purified on a G-10 Sephadex column.

### Lipopolysaccharide (LPS) dose response and time studies

Male CD-1 mice aged 6–10 weeks were weighed and given an intraperitoneal (IP) injection of 0.03, 0.3, or 3 mg/kg LPS from *Salmonella typhimurium* (Sigma Aldrich, St. Louis, MO, USA) dissolved in sterile normal saline. Mice were reweighed about 23 h after the LPS injection, anesthetized with urethane, and the jugular veins exposed. The mice were given an iv injection of ^14^C-sucrose (10^6^ dpm in 0.2 ml of lactated Ringer’s solution with 1 % BSA) 24 h ± 20 min after the LPS injection. Arterial blood was collected from a cut in the descending abdominal aorta. The vascular space of the brain was washed free of blood by opening the thorax, clamping the descending thoracic aorta, severing both jugular veins, and perfusing 20 ml of lactated Ringer’s solution through the left ventricle of the heart in less than 1 min. After washout, the mouse was immediately decapitated and the whole brain was removed and weighed. Serum was obtained by centrifuging the carotid artery blood for 10 min at 4000×*g*. The brains were solubilized, and the level of radioactivity in the brains and sera were determined in a beta counter.

A subset of mice that received 3 mg/kg of LPS was studied for ^14^C-sucrose permeation 4 h after the LPS injection. Another subset of mice that were studied for ^14^C-sucrose permeation 24 h after receiving 3 mg/kg of LPS had their brains dissected into the olfactory bulb, frontal cortex, parietal cortex, occipital cortex, striatum, hippocampus, hypothalamus, thalamus, cerebellum, pons-medulla, and midbrain.

Other mice received an injection of I-Alb instead of ^14^C-sucrose, but were otherwise treated the same, receiving 3 mg/kg of LPS and being studied at 24 h. Levels of radioactivity in the serum and brain were determined in a gamma counter.

Results were calculated by dividing the counts per minute per brain or brain region by the counts per minute per microliter in the corresponding serum and by the weight of the brain or brain region. The results were thus expressed as microliters per gram of brain tissue. Blood levels were calculated as the percent of the injected dose present per milliliter of serum (% injection/ml) by dividing the counts per minute per milliliter by the cpm injected iv and multiplying by 100.

### Administration of NAC or indomethacin

In the NAC study, male CD-1 mice were assigned to one of four groups. All mice received injections of 100 μl of saline at −30 min, 0, 6, and 24 h. In groups 1 and 3, the −30-min injection was saline, and in groups 2 and 4, it was saline containing NAC (100 mg/kg; Sigma Aldrich, St. Louis, MO). In groups 1 and 2, the remaining injections (0, 6, 24 h) were saline, whereas in groups 3 and 4, the saline vehicle contained LPS (3 mg/kg). Four hours after the last injection, mice received an injection of 10^6^ cpm of ^14^C-sucrose and were studied as described above.

Indomethacin (5 mg/kg) was studied in the same design using it in place of NAC. For the first injection, 7 % NaHCO3 was used as the vehicle instead of saline.

### Cytokine measurements

Whole brain lysates from mice treated with the three-injection regimen of LPS described above were gently homogenized in 150 μl of NP40 buffer (Invitrogen, Grand Island, NY). Samples were centrifuged for 5 min at 4 °C and 12,000×*g*. Protein content of the supernatant was then determined according to the BCA assay protocol supplied by the manufacturer. Protein concentrations are attached. A panel of 23 cytokines (interleukin (IL)-1α; IL-1β; IL-2; IL-3; IL-4; IL-5; IL-6; IL-9; IL-10; IL-12(p40); IL-12(p70); IL-13; IL-17; eotaxin (CCL11); granulocyte colony-stimulating factor (G-CSF); granulocyte-macrophage colony-stimulating factor (GM-CSF); interferon (IFN)-γ; keratinocyte chemoattractant (KC) (CXCL1); monocyte chemoattractant protein (MCP)-1 (CCL2); macrophage inflammatory protein (MIP)-1α (CCL3); MIP-1β (CCL4); regulated on activation, normal T cell expressed and secreted (RANTES; CCL5) and tumor necrosis factor (TNF)-α) were measured in whole brain lysates using a murine Bio-Plex Pro™ assay kit (Bio-Rad Laboratories, Inc.; Hercules, CA). All samples were diluted 1:3 in sample diluent provided in the kit and processed according to the manufacturer’s protocol. Plates were read on a Bio-Plex 200 (Bio-Rad Laboratories, Inc.; Hercules, CA).

### Immunohistochemistry

Control mice or mice treated with the LPS ± indomethacin regimen described above were euthanized, and their brains removed and fixed in 10 % neutral-buffered formalin, paraffin-embedded, and sectioned at 5 μm. Sections were de-paraffinized, blocked in 2 % goat serum, and exposed to 2–5 μg/ml of mouse anti-F4/80 (Serotec) or rabbit anti-Iba1 (Wako). Sections were then rinsed in Tris-buffered saline and exposed to 5 μg/ml of biotinylated anti-mouse or anti-rabbit IgG and then to Vectastain® avidin-biotin complex (ABC) kit (Vector) in conjunction with 3,3′-diaminobenzidine (DAB, Vector) to derive an insoluble stain that could be quantified.

For quantification, a minimum of two digital images per mouse cerebral cortex were taken at ×20 magnification. The images were processed in ImageJ, and the brightness and contrast adjusted before converting to an RGB stack image. The threshold of the blue stack image is adjusted until the stained tissue shows optimal contrast with highlighting of the DAB stain in red. The staining area and density were measured, and the result was expressed as an area fraction, which is the percent of the enclosed area that is highlighted in red. Relative staining intensity was then calculated for the cerebral cortices for each group of mice.

### Western blotting for ZO-1

Individual brain extracts of equivalent total protein content were resolved by sodium dodecyl sulfate-polyacrylamide gel electrophoresis (SDS-PAGE) (50 μg total protein/lane) under reducing conditions, transferred to nitrocellulose, and probed with 1 μg/ml of rabbit polyclonal antibodies to ZO-1 (Invitrogen). Blots were then probed with anti-rabbit horseradish peroxidase-conjugated secondary antibody (Jackson ImmunoResearch, West Grove, PA) (1 μg/ml) and visualized by enhanced chemiluminescence (GE Healthcare LifeSciences, Piscataway, NJ). Densities of the bands were quantified using ImageJ.

### Isolation of primary brain microvascular endothelial cells (BMECs)

Primary brain microvascular endothelial cells (BMECs) were isolated from 8-week-old CD-1 mice according to existing protocols [28, 29] with some modifications. BMECs were cultured in BMEC medium, consisting of Dulbecco’s modified Eagle’s medium (DMEM)/F12 supplemented with 20 % plasma-derived fetal bovine serum (Animal Technologies), 1 % GlutaMAX (Life Technologies), basic fibroblast growth factor (bFGF, 1 ng/ml; Roche Life Sciences), heparin (100 μg/ml), insulin (5 μg/ml), transferrin (5 μg/ml), selenium (5 ng/ml) (insulin-transferrin-selenium medium supplement; Life Technologies), and gentamicin (50 μg/ml; Sigma Aldrich). Puromycin (4 μg/ml; Sigma Aldrich) is added to the BMEC medium for the first 48 h after plating to remove pericytes and increase endothelial cell purity [30]. Cultures were maintained at 37 °C in a humidified atmosphere of 5 % CO_2_/95 % air. The medium is changed 24 h after plating to remove non-adhering cells, red blood cells, and debris. At 48 h after plating, the medium was changed again with a new medium containing all the components listed above, except puromycin. The purified primary BMECs were used to construct in vitro models when 80 % confluent, typically the 5th day after isolation.

### Immortalized mouse brain pericytes

Immortalized mouse brain pericytes (ImPCs) were prepared as previously described [31]. ImPCs were grown on uncoated flasks in low-glucose DMEM supplemented with 2 mM L-glutamine, 10 % fetal calf serum, 1 % non-essential amino acids (Life Technologies), and interferon gamma (5.6 ng/ml). ImPCs have been previously characterized [31] and stain positively for α-smooth muscle actin, CD-13, and platelet-derived growth factor receptor β and were negative for factor VIII. ImPCs were used at passages 13–15.

### Isolation of primary astrocytes

Mouse cerebral astrocytes were obtained from neonatal CD-1 mice. The meninges were carefully removed from cortices using a dissecting microscope. Then, the cleaned cortices were mechanically dissociated in astrocyte culture medium. Astrocytes were grown on poly-L-lysine-coated 24-well plates in high glucose DMEM (Life Technologies) supplemented with 10 % fetal bovine serum.

### Construction of in vitro blood-brain barrier model

BMECs were cultured alone (monocultures), co-cultured with either pericytes or astrocytes, or tri-cultured with pericytes and astrocytes according to previously published methods [32]. The triple-culture method was adjusted for co-culture and endothelial cell monoculture variants. In brief, immortalized pericytes (5000 cells/well), if present, were seeded on the bottom of a polyester Transwell insert (0.33 cm^2^, 0.4-μm pore size; Corning Inc.) and allowed to adhere for 4 h. Endothelial cells were briefly trypsinized and seeded on the inside of the Transwell insert, which was coated with fibronectin and collagen type IV (0.1 mg/ml, each), at a density of 4 × 10^4^ cells per well. The inserts were then placed in a 24-well plate containing primary astrocytes. The medium used to plate the cells in the 24-well Transwells contained all the components of the BMEC medium, listed above, with the addition of 500 nM hydrocortisone to reinforce tight junctions [33]. The medium in the luminal chamber (or the inside of the Transwell insert) was changed 24 h after seeding. BMEC monolayers were cultured for 3 days before use in experiments.

An EVOM volt ohmmeter equipped with a STX-2 electrode (World Precision Instruments; Sarasota, FL) was used to measure transendothelial electrical resistance (TEER, in ohms per square centimeter). The TEER of cell-free Transwell-clear inserts was subtracted from obtained values. TEER was measured prior to treatment and 24 h after treatment. The TEER values reported in all figures are the TEER post-treatment.

### Treatment with indomethacin and LPS

Cells were pre-treated with 50 μl BMEC + HC with or without indomethacin (200 μM). After 30 min, 50 μl BMEC + HC with or without LPS (20 μg/ml) was added to wells. Cells were treated for 24 h (indomethacin final concentration: 100 μM, LPS final concentration: 10 μg/ml).

### Transendothelial permeability

For permeability experiments, Transwell inserts were washed with serum-free BMEC + HC medium +0.1 % BSA. The same medium was added to the wells of a new 24-well plate, and the washed inserts were placed in this plate. To initiate transport experiments, ^14^C-sucrose (10^6^ cpm/ml) in medium was added to the luminal chamber. For other experiments, ^99m^Tc-albumin (10^6^ cpm/ml) was added to the loading solution in addition to the ^14^C-sucrose. Samples (500 μl) were collected from the abluminal chamber at 10, 20, 30, and 45 min, and when samples were removed, an equal volume of fresh 1 % BSA/serum-free BMEC + HC medium was immediately added to the abluminal chamber. If the loading solution only contained ^14^C-sucrose, liquid scintillation fluid was added to each sample and the radioactivity was measured using a liquid scintillation counter. If the loading solution contained ^99m^Tc-Alb and ^14^C-sucrose, each sample was acid precipitated with 30 % trichloroacetic acid and centrifuged at 5500×*g* for 10 min at 4 °C. The pellet was counted in a gamma counter for presence of ^99m^Tc. The supernatant was removed and placed into scintillation vials. Liquid scintillation fluid was added to the supernatant samples after 3 days, allowing for any unbound ^99m^Tc to decay, and counted in a beta counter.

The permeability coefficient and clearance of radioactivity was calculated according to previously published methods [34]. Clearance was expressed as microliters of radioactive tracer diffusing from the luminal to abluminal chamber and was calculated using the initial amount of radioactivity in the loading chamber and the measured amount of radioactivity in the collected samples.

$$ \mathrm{Clearance}\ \left(\upmu \mathrm{l}\right) = {\left[\mathrm{C}\right]}_{\mathrm{C}}\times {V}_{\mathrm{C}}/\ {\left[\mathrm{C}\right]}_{\mathrm{L}}, $$

where [C]_L_ is the initial amount of radioactivity per microliter of the solution loaded into the insert (in counts per minute per microliter), [C]_C_ is the radioactivity per microliter in the collected sample (in counts per minute per microliter), and *V*_C_ is the volume of collecting chamber (in microliters). The clearance volume increased linearly with time. The volume cleared was plotted versus time, and the slope was estimated by linear regression analysis. The slope of clearance curves for the BMEC monolayer plus Transwell membrane was denoted by *PS*_app_, where *PS* is the permeability × surface area product (in microliters per minute). The slope of the clearance curve with a Transwell membrane without BMECs was denoted by *PS*_membrane_. The real *PS* value for the BMEC monolayer (*PS*_e_) was calculated from

$$ 1\ /P{S}_{\mathrm{app}} = 1\ /P{S}_{\mathrm{membrane}} + 1\ /P{S}_{\mathrm{e}}. $$

The *PS*_e_ values were divided by the surface area of the Transwell inserts to generate the endothelial permeability coefficient (*P*_e_, in microliters per minute per square centimeter).

### Immunohistochemistry for BMEC

BMECs grown on Transwell inserts were washed in phosphate-buffered saline (PBS) and fixed with 4 % PFA for 10 min at 4 °C. Cells were permeabilized with 0.1 % TRITON-X100, blocked with 5 % BSA, and then incubated with anti-ZO-1 rat monoclonal antibody (Millipore, St. Charles, MO, USA), claudin-5 antibody (Abcam, Cambridge, MA), or occludin antibody (Abcam) followed by incubation with Alexa Fluor-568-conjugated secondary antibody (Invitrogen, Grand Island, NY, USA ). Inserts were mounted in antifade medium containing DAPI (nuclear) counterstain (Sigma Aldrich) and photographed with a Nikon ECLIPSE E800 fluorescence microscope.

### Soluble TREM2 ELISA

To quantify the concentrations of soluble triggering receptor expressed on myeloid cells 2 (TREM2) extracellular domain (sTREM2) in mouse EDTA-plasma samples, we adapted an anti-TREM2 ELISA system similar to that reported by Kleinberger et al. [35-37]. For the detection of sTREM2, plates were incubated overnight with polyclonal sheep anti-TREM2 capture antibody (0.25 μg/ml) (R&D Systems, Minneapolis, MN). The following day plates were washed three times with washing buffer (0.05 % Tween 20 in PBS) and blocked in 5 % BSA and 0.05 % Tween 20 in PBS (pH 7.4) for 2 h at room temperature. After blocking, the plates were washed twice and loaded with 10 μl plasma into 90 μl blocking solution and incubated overnight (4 °C). A recombinant mouse TREM2 protein (Life Technologies, Grand Isle, NY) was diluted in assay buffer in a twofold serial dilution and used for the standard curve with a concentration range of 1000, 500, 250, 125, 62, 31, 15, and 0 pg/ml. The next day, samples were removed and plates were washed three times for 5 min with washing buffer before incubation for 2 h at room temperature with mouse monoclonal anti-TREM2 antibody (1 μg/ml) (Santa Cruz Biotechnology; B-3) diluted in blocking buffer. After three additional washing steps, plates were incubated with anti-mouse HRP-conjugated secondary antibody (1:4000) and incubated for 1 h before being washed three times. Following a 30-min incubation with tetramethylbenzidine, plates were analyzed by measuring absorbance at 620 nm. Standard curve linearity and interplate and interday variability for the sTREM2 ELISA was determined using dedicated plasma sample anchors for all plates. Repeated freeze-thaw cycles had no effect on sTREM2 concentrations in plasma as previously reported [37]. The specificity of the used ELISA system was further validated by anti-TREM2 immunoblotting showing a high degree of correlation between the ELISA readings and immunoreactivity on the immunoblot using these antibodies and an independent anti-TREM2 antibody, mouse anti-TREM2 2B5 (R&D Systems, Minneapolis, MN). The plasma was stored at −70 °C until used.

### Statistical analysis

All statistical analyses and graphs were generated using GraphPad Prism® 5.0 (GraphPad Software, Inc., La Jolla, CA). Error bars represent the standard error of the mean (SEM). Comparison of two means was made using Student’s *t* test. Comparison of more than two groups was made using one-way analysis of variance (ANOVA) followed by Newman-Keuls post-test.

## Results

### Effect of LPS on BBB permeability (single injection)

**Fig. 1:**
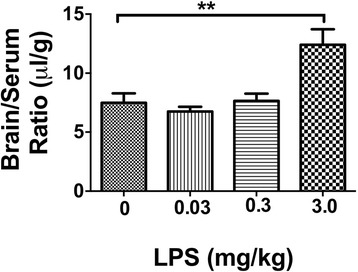
Dose-dependence of LPS on BBB permeability to ^14^C-Sucrose. Only the 3 mg/kg dose increased BBB permeability. **p < 0.01, *n* = 9/group

**Fig. 2:**
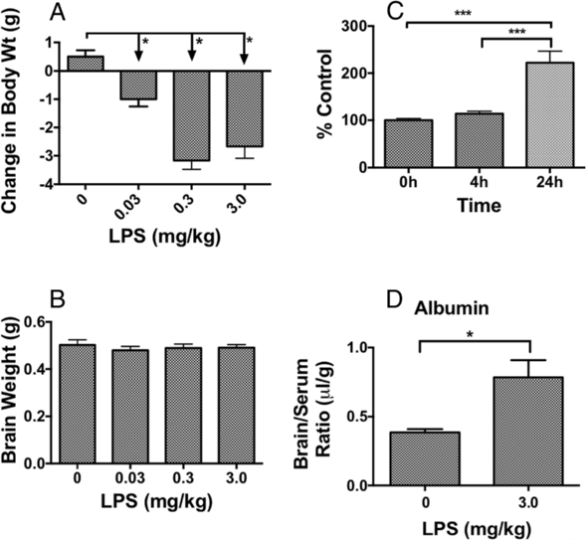
Effects of LPS on body weight, brain weight, and BBB permeability. Panel **a** shows that all doses of LPS had a significant effect on body weight (*n* = 6/group). Panel **b** shows no dose of LPS had an effect on brain weight (*n* = 6/group). Panel **c** shows that BBB permeability was increased to ^14^C-sucrose 24 but not 4 h after 3 mg/kg of LPS (*n* = 3–4/group). Panel **d** shows that 3 mg/kg LPS increased BBB permeability to albumin (*n* = 3/group). *p < 0.05; ***p < 0.001

Mice were studied 24 h after a single IP injection of LPS. The highest dose of LPS (3 mg/kg) administered to CD-1 mice produced a significant increase in BBB permeability as measured with ^14^C-sucrose (Fig. 1), whereas the lower two doses were without effect. There was a significant decrease in the amount of ^14^C-sucrose in the serum (12.4 ± 3.9 % injection/ml to 8.4 ± 3.4 % injection/ml), indicating an increased leakage of peripheral capillary beds. In contrast to effects on BBB permeability, all doses of LPS produced a decrease in body weight (Fig. 2a). No dose of LPS had an effect on brain weight (Fig. 2b). The increase in ^14^C-sucrose was evident 24 h, but not 4 h, after a dose of 3 mg/kg of LPS (Fig. 2c). LPS also increased BBB permeability to albumin (Fig. 2d). The effect of LPS differed among brain regions (Table 1). Eight of 11 brain regions showed a statistically significant increase in BBB permeability to ^14^C-sucrose, but the hypothalamus, olfactory bulb, and occipital cortex had arithmetic increases that did not reach statistical significance (Table 1). All other studies used the three-injection regimen of LPS.

**Table 1:** Effects of LPS on BBB disruption by brain region. Mice were studied 24 h after a 3 mg/kg dose of LPS given IP. Means ± SEM

Region	Control (*n* = 9; in units of μl/g)	LPS (*n* = 10; in units of μl/g)	% Change	Significance^a^
Olfactory bulb	34.5 ± 3.4	43.5 ± 3.8	26	t
Frontal cortex	6.6 ± 0.8	10.3 ± 1.0	55	*
Occipital cortex	11.5 ± 0.9	13.9 ± 1.2	20	NS
Parietal cortex	9.2 ± 0.6	12.5 ± 0.8	36	**
Striatum	14.0 ± 0.8	19.9 ± 2.0	42	*
Midbrain	10.6 ± 0.6	15.0 ± 1.2	41	**
Thalamus	8.6 ± 0.5	13.4 ± 0.9	56	***
Cerebellum	9.4 ± 0.6	14.3 ± 1.2	52	**
Pons-medulla	12.2 ± 0.6	18.8 ± 1.4	54	***
Hippocampus	10.0 ± 0.6	15.0 ± 1.2	50	**
Hypothalamus	27.2 ± 2.9	30.5 ± 3.8	12	NS

^a^Significance: NS = not significant; t = 0.1 > *p* > 0.05; *p < 0.05; **p < 0.01; ***p < 0.001

### Effect of NAC and indomethacin on LPS-induced BBB permeability

**Fig. 3:**
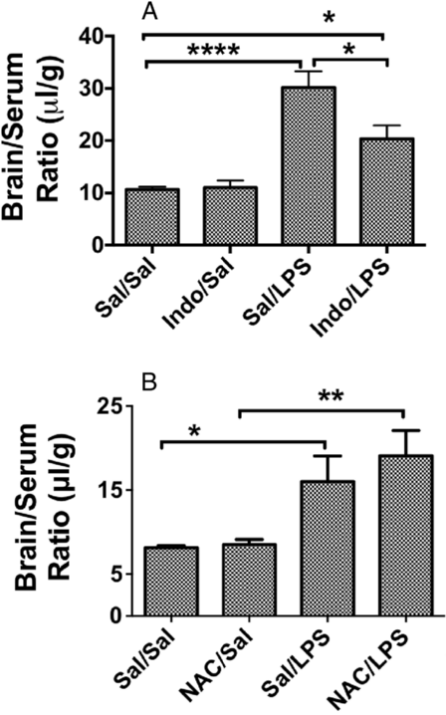
Effects of indomethacin and N-acetylcysteine on LPS-induced BBB disruption. Panel **a** shows that pre-treatment with indomethacin prevented LPS-induced BBB disruption (*n* = 7–8/group), whereas panel **b** shows that N-acetylcysteine was without effect (9–10/group). *p < 0.05; **p < 0.01; ****p < 0.001

To determine whether the increased BBB permeability induced by LPS could be blocked by the administration of the antioxidant NAC or the COX inhibitor indomethacin, we administered these agents intraperitoneally 30 min prior to the injections of LPS. For these studies, the three-injection regimen was used so as to match previously published BBB studies [6, 7, 38, 39]. As shown in Fig. 3, indomethacin (panel A), but not NAC (panel B), inhibited the LPS-induced increase in BBB permeability to ^14^C-sucrose. Indomethacin also prevented the LPS-induced decrease in body weight (data not shown) as has been previously reported [6]. Neither LPS nor indomethacin has an effect on ZO-1 protein levels as measured by Western blotting (data not shown).

### Effects of LPS and indomethacin on brain cytokines

The effects of LPS and indomethacin on brain cytokines were measured using a Bio-Plex cytokine assay kit. Of the 23 cytokines analyzed, only G-CSF and KC were not detectable. LPS produced a statistically significant increase in brain levels for eight of the cytokines (Table 2). Indomethacin prevented an LPS-induced increase of only two of these (IL-1β and IL-9) but further increased levels for four of them (IL-1α, IL-4, IL-6, and MIP-1). For five cytokines (IL-6, INF-γ, MIP-1α, MIP-1β, and RANTES), the LPS + indomethacin, but not the LPS-only group, had increased values as compared to controls.

**Table 2:** Effects of indomethacin on LPS-induced changes in cytokine levels in the brain. Values are means ± SEM in units of pg/mg protein (*n*)

Cytokine	Saline (3)	LPS (3)	LPS + indo (4)	Saline vs LPS	Saline vs LPS + indo	LPS vs LPS + indo
IL-1α	2.5 ± 0.3	12.7 ± 1.9	17.9 ± 1.5	**	***	* (indo increases)
IL-1β	19.7 ± 2.9	120.3 ± 39.8	34.6 ± 3.2	*	NS	*(indo decreases)
IL-2	15.8 ± 2.8	17.8 ± 2.1	27.3 ± 3.3	NS	NS	NS
IL-3	1.4 ± 0.2	2.1 ± 0.03	2.6 ± 0.16	*	**	NS
IL-4	0.34 ± 0.02	0.92 ± 0.08	1.1 ± 0.05	***	***	* (indo increases)
IL-5	0.93 ± 0.07	1.2 ± 0.16	1.1 ± 0.1	NS	NS	NS
IL-6	1.5 ± 0.12	29.7 ± 16.5	72.8 ± 13.1	NS	*	* (indo increases)
IL-9	75.0 ± 3.1	156.9 ± 16.4	89.2 ± 7.0	**	NS	**(indo decreases)
IL-10	6.3 ± 0.1	9.0 ± 0.3	10.4 ± 0.6	**	**	NS
IL-12(p40)	8.5 ± 1.9	1084 ± 648.4	385.2 ± 81.8	NS	NS	NS
IL-12(p70)	72.5 ± 6.5	102.1 ± 3.1	97.6 ± 10.3	NS	NS	NS
IL-13	38.1 ± 2.3	40.0 ± 3.5	27.7 ± 3.5	NS	NS	NS
IL-17	19.4 ± 2.3	24.8 ± 1.6	20.5 ± 0.9	NS	NS	NS
Eotaxin	2213 ± 131.5	2671 ± 99.8	2165 ± 144.3	NS	NS	NS
GM-CSF	28.6 ± 2.5	47.1 ± 1.3	43.9 ± 2.1	**	**	NS
IFN-γ	16.1 ± 2.1	20.6 ± 1.1	22.9 ± 1.3	NS	*	NS
MCP-1	33.0 ± 4.6	1550 ± 300.6	1612 ± 275.1	**	**	NS
MIP-1a	6.8 ± 0.7	128.3 ± 36.1	465.2 ± 108.4	NS	*	* (indo increases)
MIP-1b	56.6 ± 3.5	128.9 ± 15.7	190.5 ± 37.0	NS	*	NS
RANTES	1.5 ± 0.3	154.8 ± 37.7	280.1 ± 57.3	NS	**	NS
TNF-α	459.9 ± 34.3	482.1 ± 21.3	432.2 ± 16.7	NS	NS	NS

*p < 0.05; **p < 0.01; ***p < 0.001

### Microglial and astrocytic activation

**Fig. 4:**
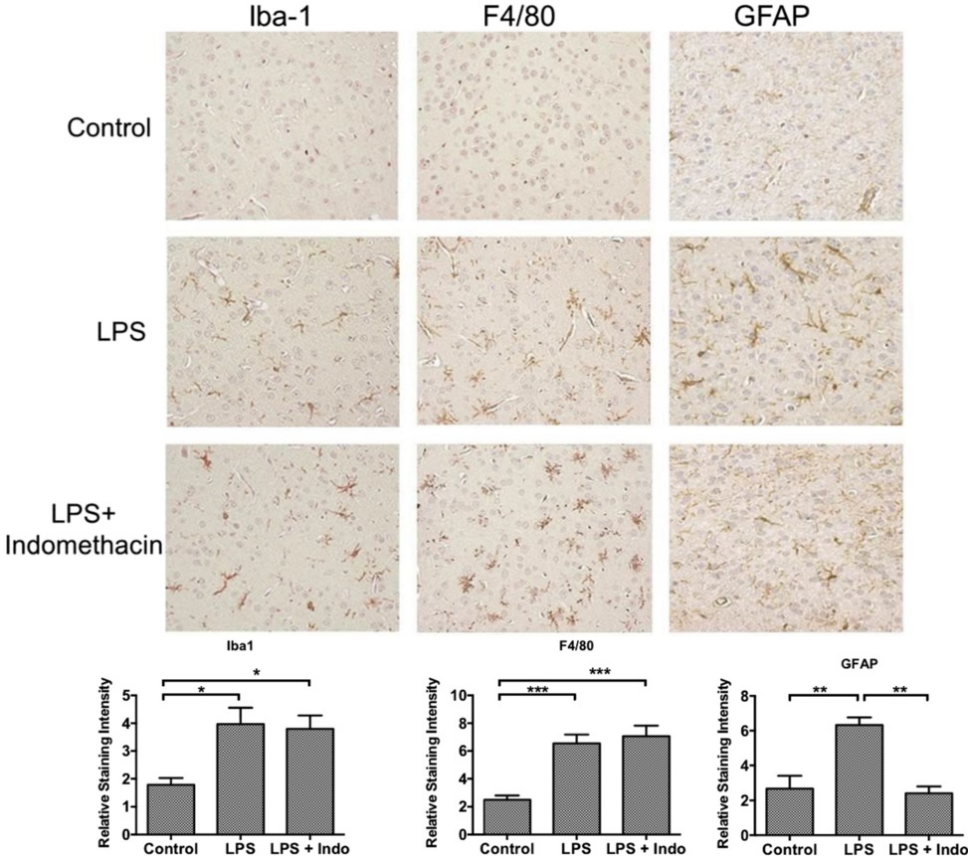
Effects of LPS and indomethacin on microglial/macrophage and astrocytic activation. Microglial/macrophage activation was increased by LPS but not blocked by indomethacin as assessed by Iba1 (*left-hand panels*) and by F4/80 (*middle panels*) immunohistochemistry. Astrocytic activation was increased by LPS and blocked by indomethacin as assessed by GFAP staining (*right-hand panels*). The *bottom three panels* show quantification of Iba1 (*n* = 5–6/statistical cell), F4/80 (*n* = 5–6), and GFAP (*n* = 4–5) immunohistochemical staining. *p < 0.05, **p < 0.01, ***p < 0.001, Mag = ×20

Figure 4 shows the results of immunohistochemical staining for microglia/macrophages (Iba1 and F4/80) and astrocytes (glial fibrillary acidic protein (GFAP)). The lower panels of Fig. 4 show the quantification of Iba1, F4/80, and GFAP staining. LPS increased staining for Iba1 and F4/80 staining, an effect not blocked by indomethacin. LPS also increased staining for GFAP, but this effect was blocked by indomethacin.

### Peripheral macrophage/monocyte activation

**Fig. 5:**
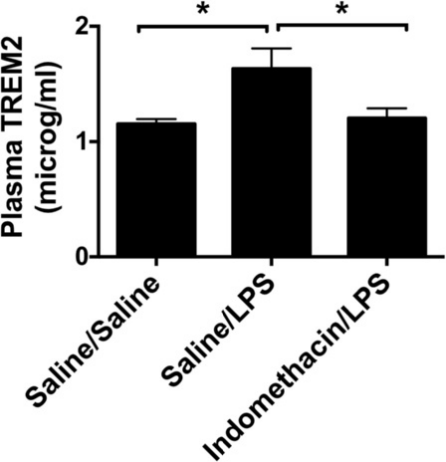
Effects of LPS plasma TREM2. Plasma TREM2 levels are increased by LPS, an effect blocked by treatment with indomethacin (*n* = 8/group). *p < 0.05

Figure 5 shows the effects of LPS and indomethacin on plasma levels of TREM2, a cell surface signaling molecule for macrophages/monocytes that regulates proinflammatory responses and phagocytosis [40, 41] and is increased with LPS treatment [42]. LPS induced an increase in levels of plasma TREM2 that was blocked by indomethacin.

### LPS and indomethacin effects on in vitro monolayers of BMECs

**Fig. 6:**
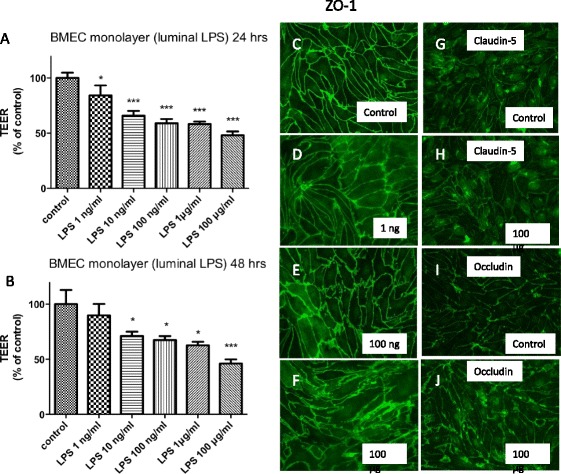
Effects of LPS on TEER of BMECs and cytoarchitecture of ZO-1, claudin-5, and occludin. Panels **a** and **b** show that LPS added either 24 or 48 h earlier to monocultures of BMECs decreased their TEER in a dose-dependent manner, consistent with disruption of the BBB. Immunostaining for ZO-1 shows increasing dose-dependent cytoarchitectural disorganization for ZO-1 (panels **c–f**). Immunostaining for claudin-5 (panels **g** and **h**) does not clearly show such disorganization, whereas immunostaining for occludin does (panels **i** and **j**). *p < 0.05; ***p < 0.001

**Fig. 7:**
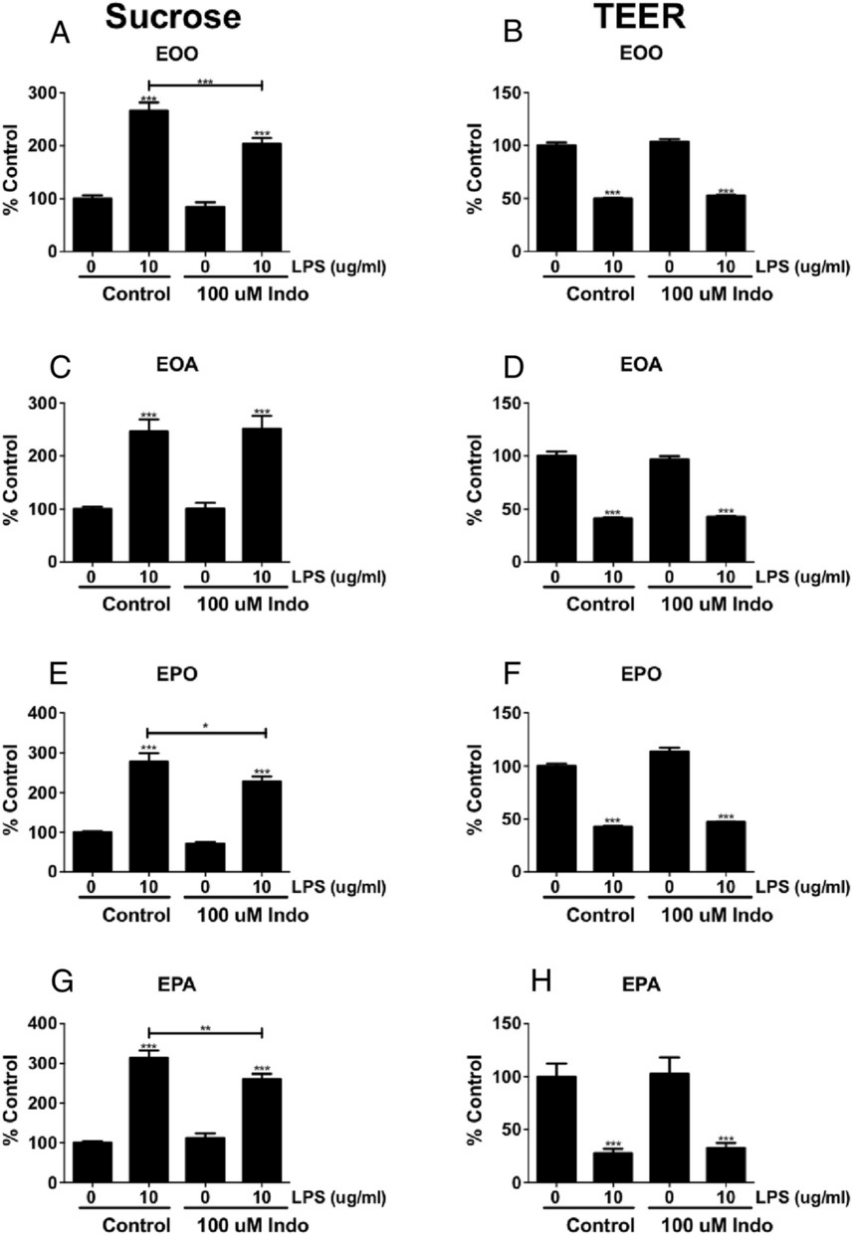
Effect of LPS and indomethacin (*Indo*) on TEER and ^14^C-sucrose permeability in monolayers of BEC monocultures (*EOO*, *n* = 10), BEC + astrocyte co-cultures (*EOA*, *n* = 5), BEC + pericyte co-cultures (*EPO*, *n* = 5), and BEC + pericyte + astrocyte tri-cultures (*EPA*, *n* = 10). Panel **a** shows that in monocultures of BECs, LPS increased permeability to sucrose and that indomethacin partially blocked this effect. Panel **b** shows that LPS increased permeability of monolayer monocultures of BEC as measured by TEER and that indomethacin had no effect on LPS-induced permeability. Co-cultures of BECs with astrocytes (panels **c** and **d**) or pericytes (panels **e** and **f**) or tri-cultures of BECs with pericytes and astrocytes (panels **g** and **h**) did not produce results substantially different from those obtained with monocultures of BECs. *Y*-axis is *%Control* of Pe in units of microliters per minute per square centimeter. *p < 0.05, ***p < 0.001; *without bar* compares to respective 0 value; *with bar* compares LPS 10 μg/ml without indomethacin to LPS 10 μg/ml with indomethacin

**Fig. 8:**
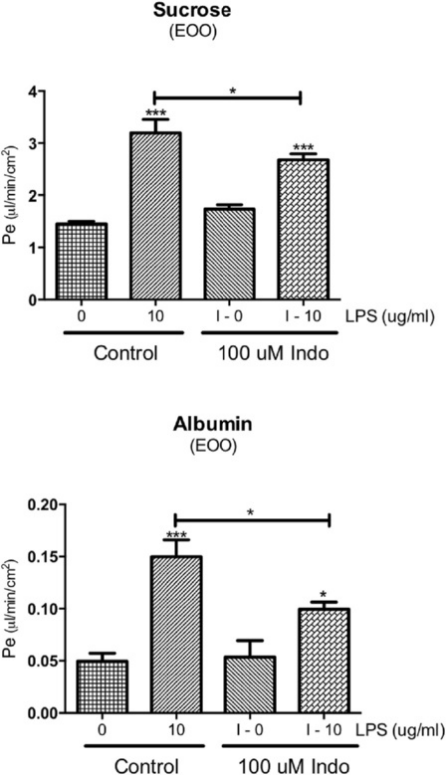
Effect of LPS and indomethacin on ^14^C-sucrose (*upper panel*) and Tc-Alb permeabilities (*lower panel*) in monocultures of BECs. Results show that indomethacin partially blocked the LPS-induced permeability for both sucrose and albumin. *p <0.05, ***p <0.001; without bar compares to respective 0 value; with bar compares indicatedgroups

All studies were done in duplicates, and to reduce interday variation, results were expressed as percent of control. Figure 6a, b show that LPS had a dose-dependent response on TEER after 24 and 48 h of exposure. Figure 6c–f show a dose-dependent effect on the cytoarchitecture of the tight junction protein ZO-1, with higher concentrations of LPS producing increasing amounts of ZO-1 protein distributed away from the edges of the brain endothelial cells resulting in a hazy appearance in the cytoplasm. LPS produced a similar altered cytoarchitecture for occludin (Fig. 6i, j) but not for claudin-5 (Fig. 6g, h). LPS applied to monocultures of BECs induced BBB disruption as shown by increased ^14^C-sucrose penetration (Fig. 7a) and increased TEER (Fig. 7b). Indomethacin partially blocked the LPS-induced BBB disruption to ^14^C-sucrose but had no effect on disruption as measured by TEER. Co-cultures of BECs and astrocytes (Fig. 7c, d) or BECs and pericytes (Fig. 7e, f) or tri-cultures of BECs, pericytes, and astrocytes (Fig. 7g, h) did not produce results different from those of a monoculture of BECs. Figure 8 shows that LPS also induced disruption to Tc-Alb in BEC monocultures and that this disruption was partially blocked by indomethacin.

## Discussion

Inflammation, including inflammation induced by LPS, has long been known to disrupt the BBB [3, 4, 43]. However, many aspects of inflammation-induced BBB disruption remain unexplored. Here, we examined mechanisms that underlie inflammation-related BBB disruption, beginning with a characterization of that disruption. We found that in comparison to induction of weight loss, a high dose of LPS was required to induce in vivo BBB disruption. Specifically, 3 mg/kg, but not 0.3 nor 0.03 mg/kg, disrupted the BBB. In contrast, all doses of LPS, including the very low dose of 0.03 mg/kg, induced loss of body weight. By this measure, the BBB is resistant to LPS-induced disruption in that a relatively high dose was needed to induce disruption. This shows that LPS can activate neuroimmune pathways and processes such as sickness behavior without inducing or being dependent on BBB disruption.

In comparison to the relative sensitivity to body weight loss, brain weight was not affected even by the dose that disrupted the BBB. This is not altogether expected as brain disruption underlies vasogenic edema; therefore, a disruption of the BBB, if prolonged, would be expected to lead to brain edema and, hence, an increase in brain weight.

BBB disruption as measured by the increase in sucrose space was not uniform throughout the brain but varied among brain regions. The hypothalamus, olfactory bulb, and occipital cortex had small increases that did not reach statistical significance, whereas the thalamus, frontal cortex, cerebellum, and pons-medulla had increases of over 50 %. This pattern differs from other causes of disruption. For example, in diabetes mellitus, BBB disruption first occurs in the midbrain with the cerebellum being an area resistant to diabetes-induced BBB disruption [44]. This shows that regional vulnerabilities exist among regions of the BBB but suggests that those regions vary as a function of insult.

We found the BBB was disrupted to both albumin and sucrose. BBB integrity is induced by three modifications of the capillary bed: an induction of tight junctions that prevents intercellular (paracellular) leakage, a decrease in macropinocytosis, and a loss of intracellular pores and fenestrae. Disruption of the BBB can be caused by reversal of any or all of these modifications. Sucrose, with a molecular weight of 342 Da, is the smallest of the classical vascular markers and thus is the most sensitive for detecting BBB disruption. Albumin at about 65 kDa is the largest of the classical vascular markers used to measure BBB disruption and thus requires a major disruption for detection. TEER, which can be used in vitro, requires a patent channel for the exchange of electrolytes between the luminal and abluminal chambers. A small amount of sucrose crosses the BBB independently of BBB disruption by the mechanism of transcellular diffusion, and a small amount of albumin enters the CNS primarily through the extracellular pathways [45]. Hence, control values for neither sucrose nor albumin are zero, and the values obtained here in control mice are those expected based on their entry rates of about 10^−4^ ml/g-min for sucrose and 10^−5^ to 10^−6^ ml/g-min for albumin. Here, we found significant disruptions of the BBB as assessed by both sucrose and albumin in vivo and by TEER, sucrose, and albumin in vitro. Previous work has suggested that BBB disruption to smaller molecules, but not larger molecules, is indicative of opening of the paracellular route only, whereas disruption to larger and smaller molecules is suggestive of opening of both the paracellular and the transcellular routes [17, 18]. Cytoarchitectural translocation of the tight junction proteins ZO-1 and occludin also support an increase in paracellular permeability. Claudin-5 did not show a change with LPS treatment, but this may be because it is the most deeply embedded of these tight junction proteins [46] so that it would be the last to be translocated in a sequential disassembly of tight junctions. Taken together, the in vivo studies finding disruption to both sucrose and to albumin and the in vitro studies assessing TEER, tight junction protein analysis, and the permeability to sucrose and to albumin suggest that LPS results in disruption by both the paracellular and the transcellular pathways.

We found that BBB disruption was present 24 h after injection of a single dose of 3 mg/kg of LPS, but not 4 h after the injection. A recent study has characterized the effect of a similar regimen of LPS on the levels of 13 cytokines in the brain and blood and can be used to indicate which cytokines are less likely to be involved in BBB disruption [47]. It is likely that those brain cytokines not elevated at either 4 or 24 h (IL-1α, IL-13, IP-10, KC, TNF-α, GM-CSF) are less likely to be involved in disruption than those elevated at either 4 or 24 h (IL-1β, IL-6, IL-10, G-CSF, RANTES, MCP-1, MIP-1α). Likewise, those brain cytokines found here in which the indomethacin + LPS group did not differ from the LPS-only group (Il-2, Il-3, Il-5, Il-10, Il-12(p40), Il-12(p70), Il-13, Il-17, eotaxin, TNF, GM-CSF, interferon-gamma, MCP-1, MIP-1β, RANTES) are unlikely to be involved in mediating BBB disruption.

We then determined the effects of the antioxidant N-acetylcysteine (NAC) and the nonsteroidal anti-inflammatory drug indomethacin on LPS-induced BBB disruption. NAC rapidly crosses the BBB [27, 48], protects BEC from oxidative stress induced by methamphetamine and antiretroviral drugs [25, 26, 48], and reverses the decreased brain-to-blood transport of amyloid beta peptide induced by LPS [27]. Indomethacin as a COX inhibitor decreases prostaglandin, prostacyclin, and thromboxane syntheses and allows through mass action an increase in leukotrienes [49]. It also reverses the effect of LPS on amyloid beta peptide efflux [23] and potentiates LPS-enhanced transport of gp120 across the BBB [50] but is without effect on LPS-induced inhibition of P-glycoprotein function [24]. Here, we found that NAC was without effect but that indomethacin prevented LPS-induced BBB disruption to ^14^C-sucrose.

Indomethacin acts by blocking COX activity, thus reducing prostaglandin production. This strongly indicates that LPS induces BBB disruption through a COX-dependent pathway, possibly one that involves secretion of cytokines from the BECs. Release of prostaglandins from BECs is known to mediate the fever induced by LPS, and indomethacin attenuates that fever by blocking prostaglandin synthesis [51, 52].

That LPS is acting directly on BECs to induce disruption and that no other cell type is required for LPS to produce disruption under the conditions of these experiments are clearly shown in the in vitro studies. As shown in Fig. 6, LPS disrupted monolayers of BECs; no cell other than BECs was incubated in those monolayers. Therefore, LPS must be able to disrupt the BBB by acting directly on BECs. LPS is also known from other studies to induce neuroinflammation [53]; we also saw LPS-induced neuroinflammation here as evidenced by increases in Iba1, GFAP, and F4/80 immunostaining and in brain levels of eight cytokines. Therefore, it is reasonable to assume that other components of the neurovascular unit, such as astrocytes or pericytes, would be involved in promoting the LPS-induced disruption of the BBB. However, we found that adding neither pericytes nor astrocytes altered the response of BECs to LPS. This suggests that BECs alone are involved in BBB disruption in our in vitro model, although it is still possible that other cells contribute to neuroinflammation-induced BBB disruption under other experimental conditions. In comparison to LPS-induced disruption of the BBB, LPS-induced enhancement of insulin transport across the BBB involves nitric oxide release from non-BBB cells [54] and LPS-enhanced transport of HIV-1 is enhanced by the presence of pericytes [50].

Indomethacin had mixed effects on LPS-induced inflammation. Indomethacin did decrease the LPS-induced elevation in brain levels of IL-1β, IL-9, and plasma TREM2, the latter used as a measure of peripheral inflammation. Indomethacin decreased the LPS-induced increase in GFAP, suggesting a role for COX in astrocytic activation, but had no effect on the LPS-induced increase in Iba1 or F4/80 staining, suggesting that activation of microglia/macrophages is independent of COX. Additionally, indomethacin further increased over LPS alone the levels of four other cytokines.

Likewise, indomethacin had a mixed effect on BBB disruption as measured in vitro, having no effect on disruption as measured by TEER, but partially blocking disruption as measured by sucrose and albumin. BBB disruption as measured by TEER is more associated with paracellular pathways, whereas BBB disruption as measured by sucrose and albumin is associated with transcytosis. This suggests that although LPS induces disruption by both paracellular and transcytotic pathways, the paracellular pathway is mediated through COX-independent mechanisms.

There are several implications of this work. First, although LPS is not a true sepsis model, it does suggest that COX inhibition would be protective against the BBB disruption seen in sepsis and in other inflammatory states. Additionally, it is increasingly clear that LPS enters the circulation not only in disease states such as the leaky gut of AIDS [55] but also under more physiologic conditions, such as after ingestion of a high-fat meal or marathon running [56, 57]. The latter studies suggest that LPS may have roles in neuroimmune modulation that are not strictly pathological. For example, LPS is likely one of the mediators responsible for the differential response of tissues, including the brain, to healthy and pathologic microbiomes [58, 59]. These studies found that high doses of LPS are required to disrupt the BBB when the LPS exposure is acute but did not investigate the effects of chronic, low-dose exposure to LPS on BBB integrity.

## Conclusions

These results show that the BBB is relatively resistant to disruption by LPS in comparison to another classic effect of LPS, that of weight loss. Not all regions of the BBB are equally sensitive to LPS-induced BBB disruption, involvement of non-BECs is not required for LPS to induce disruption, and disruption occurs through both paracellular and transcytotic mechanisms with transcytotic disruption being prostaglandin-dependent.

## Abbreviations

BBB: blood-brain barrier
BECs: brain endothelial cells
BMECs: brain microvascular endothelial cells
BSA: bovine serum albumin
COX: cyclooxygenase
I-Alb: iodine-labeled albumin
IL: interleukin
ImPCs: immortalized mouse brain pericytes
LPS: lipopolysaccharide
NAC: N-acetylcysteine
Tc-Alb: technetium-labeled albumin
TEER: transendothelial electrical resistance
TREM2: triggering receptor expressed on myeloid cells 2

## References

[1] Skoog T (1937). On the vital staining of the central nervous system. Acta Otolaryngologica.

[2] Rapoport SI (1976). Pathological alterations of the blood-brain barrier. Blood-brain barrier in physiology and medicine.

[3] Allen IV (1965). The effect of bacterial pyrogen on the blood-brain barrier for trypan blue. J Pathol Bacteriol.

[4] Eckman PL, King WM, Brunson JG (1958). Studies on the blood brain barrier. I. Effects produced by a single injection of gram-negative endotoxin on the permeability of the cerebral vessels. Am J Pathol.

[5] Persidsky Y, Stins M, Way D, Witte MH, Weinand M, Kim KS (1997). A model for monocyte migration through the blood-brain barrier during HIV-1 encephalitis. J Immunol.

[6] Banks WA, Kastin AJ, Brennan JM, Vallance KL (1999). Adsorptive endocytosis of HIV-1gp120 by blood-brain barrier is enhanced by lipopolysaccharide. Exp Neurol.

[7] Xaio H, Banks WA, Niehoff ML, Morley JE (2001). Effect of LPS on the permeability of the blood-brain barrier to insulin. Brain Res.

[8] Minami T, Okazaki J, Kawabata A, Kuroda R, Okazaki Y (1998). Penetration of cisplatin into mouse brain by lipopolysaccharide. Toxicology.

[9] Pan W, Yu C, Hsuchou H, Zhang Y, Kastin AJ (2008). Neuroinflammation facilitates LIF entry into brain: role of TNF. Am J Physiol Cell Physiol.

[10] Tucsek Z, Toth P, Sosnowska D, Gautam T, Mitschelen M, Koller A, et al. Obesity in aging exacerbates blood-brain barrier disruption, neuroinflammation, and oxidative stress in the mouse hippocampus: effects on expression of genes involved in beta-amyloid generation and Alzheimer’s disease. J Gerontol A Biol Sci Med Sci. 2013, epub. 10.1093/gerona/glt177 PMC4172034 24269929

[11] Sengillo JD, Winkler EA, Walker CT, Sullivan JS, Johnson M, Zlokovic BV (2012). Deficiency in mural vascular cells coincides with blood-brain barrier disruption in Alzheimer’s disease. Brain Pathol.

[12] Starr JM, Wardlaw J, Ferguson K, MacLullich A, Deary IJ, Marshall I (2003). Increased blood-brain barrier permeability in type II diabetes demonstrated by gadolinium magnetic resonance imaging. J Neurol Neurosurg Psychiatry.

[13] Abraham CS, Harada N, Deli MA, Niwa M (2003). Transient forebrain ischemia increases the blood-brain barrier permeability for albumin in stroke-prone spontaneously hypertensive rats. Cell Mol Neurobiol.

[14] Pozzilli C, Bernardi S, Mansi L, Picozzi P, Iannotti F, Alfano B (1988). Quantitative assessment of the blood-brain barrier permeability in multiple sclerosis using 68-Ga-EDTA and positron emission tomography. J Neurol Neurosurg Psychiatry.

[15] Reese TS, Karnovsky MJ (1967). Fine structural localization of a blood-brain barrier to endogenous peroxidase. J Cell Biol.

[16] Brightman MW, Reese TS (1969). Junctions between intimately apposed cell membranes in the vertebrate brain. J Cell Biol.

[17] Mayhan WG, Heistad DD (1985). Permeability of blood-brain barrier to various sized molecules. Am J Physiology.

[18] Ziylan YZ, Robinson PJ, Rapoport SI (1984). Blood-brain barrier permeability to sucrose and dextran after osmotic opening. Am J Physiol.

[19] Knowland D, Arac A, Sekiguchi KJ, Hsu M, Lutz SE, Perrino J (2014). Stepwise recruitment of transcellular and paracellular pathways underlies blood-brain barrier breakdown in stroke. Neuron.

[20] Fleegal-DeMotta MA, Dohgu S, Banks WA (2009). Angiotensin II modulates BBB permeability via activation of the AT1 receptor in brain endothelial cells. J Cereb Blood Flow Metab.

[21] De Vries HE, Blom-Roosemalen MCM, De Boer AG, van Berkel TJ, Breimer DD, Kuiper J (1996). Effect of endotoxin on permeability of bovine cerebral endothelial cell layers in vitro. J Pharmacol Exp Ther.

[22] Minami T, Okazaki J, Kawabata A, Kawaki H, Okazaki Y, Tohno Y (1998). Roles of nitric oxide and prostaglandins in the increased permeability of the blood-brain barrier caused by lipopolysaccharide. Environ Toxicol Pharmacol.

[23] Jaeger JB, Dohgu S, Lynch JL, Fleegal-DeMotta MA, Banks WA (2009). Effects of lipopolysaccharide on the blood-brain barrier transport of amyloid beta protein: a mechanism for inflammation in the progression of Alzheimer’s disease. Brain Behav Immun.

[24] Salkeni MA, Lynch JL, Price TO, Banks WA (2009). Lipopolysaccharide impairs blood-brain barrier P-glycoprotein function in mice through prostaglandin- and nitric oxide-independent pathways and nitric oxide-independent pathways. J Neuroimmune Pharmacology.

[25] Zhang X, Banerjee A, Banks WA, Ercal N (2009). N-acetyl amide protects against methamphetamine-induced oxidative stress and neurotoxicity in immortalized human brain endothelial cells. Brain Res.

[26] Manda KR, Banerjee A, Banks WA, Ercal N (2011). Highly active antiretroviral therapy drug combination induces oxidative stress and mitochondrial dysfunction in immortalized human blood-brain barrier endothelial cells. Free Radic Biol Med.

[27] Erickson MA, Hansen K, Banks WA (2012). Inflammation-induced dysfunction of the low-density lipoprotein receptor-related protein-1 at the blood-brain barrier: protection by the antioxidant N-acetylcysteine. Brain Behav Immun.

[28] Coisne C, Dehouck L, Faveeuw C, Delplace Y, Miller F, Landry C (2005). Mouse syngenic in vitro blood-brain barrier model: a new tool to examine inflammatory events in cerebral endothelium. Lab Invest.

[29] Jacob A, Hack B, Chiang E, Garcia JG, Quigg RJ, Alexander JJ (2010). C5a alters blood-brain barrier integrity in experimental lupus. FASEB J.

[30] Perriere N, Demeuse P, Garcia E, Regina A, Debray M, Andreux JP (2005). Puromycin-based purification of rat brain capillary endothelial cell cultures. J Neurochem.

[31] Shah GN, Price TO, Banks WA, Morofuji Y, Kovac A, Ercal N (2013). Pharmacological inhibition of mitochondrial carbonic anhydrases protects mouse cerebral pericytes from high glucose-induced oxidative stress and apoptosis. J Pharmacol Exp Therap.

[32] Nakagawa S, Deli MA, Kawaguchi H, Shimizudani T, Shimono T, Kittel A (2009). A new blood-brain barrier model using primary rat brain endothelial cells, pericytes and astrocytes. Neurochem Int.

[33] Hoheisel D, Nits T, Franke H, Wegener J, Hakvoort A, Tilling T (1998). Hydrocortisone reinforces the blood-brain barrier properties in a serum free cell culture system. Biochem Biophys Res Commun.

[34] Dehouck MP, Jolliet-Riant P, Bree F, Fruchart JC, Cecchelli R, Tillement JP (1992). Drug transfer across the blood-brain barrier: correlation between in vitro and in vivo models. J Neurochem.

[35] Scimemi A, Meabon JS, Woltjer RL, Sullivan JM, Diamond JS, Cook DG (2013). Amyloid-β1-42 slows clearance of synaptically released glutamate by mislocalizing astrocytic GLT-1. J Neurosci.

[36] Daws MR, Lanier LL, Seaman WE, Ryan JC (2001). Cloning and characterization of a novel mouse myeloid DAP 12-associated receptor family. Eur J Immunol.

[37] Kleinberger G, Yamanishi Y, Suarez-Calvet M, Czirr E, Lohmann E, Cuyvers E (2014). TREM2 mutations implicated in neurodegeneration impair cell surface transport and phagocytosis. Sci Transl Med.

[38] Nonaka N, Shioda S, Banks WA (2005). Effect of lipopolysaccharide on the transport of pituitary adenylate cyclase activating polypeptide across the blood-brain barrier. Exp Neurol.

[39] Erickson MA, Hartvigson PE, Morofuji Y, Owen JB, Butterfield DA, Banks WA (2012). Lipopolysaccharide impairs amyloid beta efflux from brain: altered vascular sequestration, cerebrospinal fluid reabsorption, peripheral clearance and transporter function at the blood-brain barrier. J Neuroinflammation.

[40] Turnbull IR, Gilfillan S, Cella M, Aoshi T, Miller MC, Piccio L (2006). Cutting edge: TREM-2 attenuates macrophage activation. J Immunol.

[41] Hamerman JA, Jarjoura JR, Humphrey MB, Nakamura MC, Seaman WE, Lanier LL (2006). Cutting edge: inhibition of TLR and FcR responses in macrophages by triggering receptor expressed on myeloid cells (TREM0-2 and DAP12). J Immunol.

[42] Schmid CD, Melchior B, Masek K, Puntambekar SS, Danielson PE, Lo DD (2009). Differential gene expression in LPS/IFNgamma activated microglia and macrophages: in vitro versus in vivo. Neurochem.

[43] Wispelwey B, Lesse AJ, Hansen EJ, Scheld WM (1988). Haemophilus influenzae lipopolysaccharide-induced blood brain barrier permeability during experimental meningitis in the rat. J Clin Investig.

[44] Huber JD, VanGilder RL, Houser KA (2006). Streptozotocin-induced diabetes progressively increases blood-brain barrier permeability in specific brain regions in rats. Am J Physiol.

[45] Kim YS, Lee MH, Wisniewski HM (1986). Aluminum induced reversible change in permeability of the blood-brain barrier to [14C]sucrose. Brain Res.

[46] Brown RC, Davis TP (2002). Calcium modulation of adherens tight junction function: a potential mechanism for blood-brain barrier disruption after stroke. Stroke.

[47] Erickson MA, Banks WA (2011). Cytokine and chemokine responses in serum and brain after single and repeated injections of lipopolysaccharide: multiplex quantification with path analysis. Brain, Behavior, & Immunity.

[48] Farr SA, Poon HF, Dogrukol-Ak D, Drake J, Banks WA, Eyerman E (2003). The antioxidants alpha-lipoic acid and N-acetylcysteine reverse memory impairment and brain oxidative stress in aged SAMP8 mice. Journal of Neurochemisrty.

[49] Vane JR, Botting RM (1997). Mechanism of action of anti-inflammatory drugs. Adv Exp Med Biol.

[50] Dohgu S, Banks WA (2008). Lipopolysaccharide-enhanced transcellular transport of HIV-1 across the blood-brain barrier is mediated by the p38 mitogen-activated protein kinase pathway. Exp Neurol.

[51] Engstrom L, Ruud J, Eskilsson A, Larsson A, Mackerlova L, Kugelberg U (2012). Lipopolysaccharide-induced fever depends on prostaglandin E2 production specifically in brain endothelial cells. Endocrinology.

[52] Inoue W, Matsumura K, Yamagata K, Takemiya T, Shiraki T, Kobayashi S (2002). Brain-specific endothelial induction of prostaglandin E(2) synthesis enzymes and its temporal relation to fever. Neurosci Res.

[53] Qin L, Wu X, Block ML, Liu Y, Breese GR, Hong JS (2007). Systemic LPS causes chronic neuroinflammation and progressive neurodegeneration. Glia.

[54] Banks WA, Dohgu S, Nakaoke R, Lynch JL, Fleegal-DeMotta MA, Erickson MA (2008). Nitric oxide isoenzymes regulate LPS-enhanced insulin transport across the blood-brain barrier. Endocrinology.

[55] Brenchley JM, Price DA, Schacker TW, Asher TE, Silvestri G, Rao S (2006). Microbial translocation is a cause of systemic immune activation in chronic HIV injection. Nat Med.

[56] Ng QY, Lee KW, Byrne C, Ho TF, Lim CL (2008). Plasma endotoxin and immune responses during a 21-km road race under a warm and humid environment. Ann Acad Med Singapore.

[57] Amar J, Burcelin R, Ruidavets JB, Cani PD, Fauvel J, Alessi MC (2008). Energy intake is associated with endotoxemia in apparently healthy men. Am J Clin Nutr.

[58] Jasarevic E, Howerton CL, Howard CD, Bale TL (2015). Alterations in the vaginal microbiome by maternal stress are associated with metabolic reprogramming of the offspring gut and brain. Endocrinology.

[59] Banks WA (2015). A vagina monologue: mom’s stress, bugs, and baby’s brain. Endocrinology.

